# Real-life medium term follow-up data for intravitreal dexamethasone implant in retinal vein occlusion

**DOI:** 10.1038/s41598-021-87467-6

**Published:** 2021-04-15

**Authors:** Thomas Wecker, Bastian Grundel, Milena Grundel, Marie-Christine Bründer, Simon Trick, Clemens Lange, Daniel Böhringer, Hansjürgen Agostini, Andreas Stahl

**Affiliations:** 1 Eye Center, Medical Center, Faculty of Medicine, University of Freiburg, Freiburg, Germany; 2 Ophthalmic Practice Dr. Wecker, Heilbronn, Germany; 3 Department of Ophthalmology, University Medical Center, Greifswald, Germany; 4 Department of Ophthalmology, University Medical Center, Ulm, Germany

**Keywords:** Medical research, Retinal diseases

## Abstract

Macular edema (ME) is the most frequent vision threatening consequence after retinal vein occlusion (RVO). In this study, we evaluate the effect of dexamethasone intravitreal implants (DII, Ozurdex) in a real-life cohort of 99 patients with ME due to RVO. All patients who received DII for ME following RVO between 2011 and 2016 at the University Eye Hospital Freiburg, Germany and who had fully accessible electronic medical records were eligible for this study. Most of the patients included in this study were not treatment-naïve: 61 eyes had received prior anti-VEGF drugs, 6 eyes had received intravitreal corticosteroids (triamcinolone) and 15 had been treated with both; 17 eyes were treatment-naïve. Mean follow-up was 312 ± 310 days. Mean visual acuity (VA) was maintained throughout the observation period (mean VA at baseline: 66.7 ± 23.5 letters; at last observation 64.9 ± 28.3). Central retinal thickness (CRT) decreased from 526 ± 179 µm at baseline to 431 ± 199 µm. Mean intraocular pressure (IOP) increased from 14.4 ± 3.1 mmHg at baseline to 17.1 ± 6.3 mmHg. Cataract surgery was performed in 22% of phakic eyes. DII was used as second-line treatment in the majority of cases in this cohort. The fact that mean VA remained unchanged while mean CRT decreased illustrates that morphologic improvement does not always translate into functional gain. Mean IOP was maintained within normal limits and cataract formation was as expected in this age group.

## Introduction

Retinal vein occlusion (RVO) is the second most common retinal vascular disease after diabetic retinopathy^1^. Commonly, patients in the second half of their lives are affected. Due to this age distribution and the increase in risk factors such as arterial hypertension, diabetes and obesity due to demographical changes, the frequency of RVO has increased in recent years^2^. This trend is likely to continue in the coming years. It is currently estimated that 16.4 million adults worldwide are affected by RVO and its sequelae. The cumulative 10-year incidence of RVO was 1.6% in the Blue Mountain Eye Study cohort of patients 49 years and older^3^. As a consequence, macular edema due to RVO is a common cause of significant visual acuity (VA) loss. The available literature does not allow a reliable estimate on the incidence of ME in RVO patients probably because patients with RVO but without ME may remain asymptomatic and not present to our clinics^4^.

It is well established that if significant ischemic retinopathy and pathologic growth of blood vessels occurs as a consequence of RVO, retinal laser photocoagulation is indicated in order to avoid subsequent complications. VA loss in RVO can be a result of either ischemic maculopathy, macular edema or a combination of both. While no therapeutic approach exists to improve ischemic maculopathy, macular edema can in many cases be efficiently treated with the intravitreal application of either anti-vascular endothelial growth factor agents (anti-VEGF) or corticosteroids^5^. Chronically persistent or recurrent edema, however, poses a therapeutic challenge and the published literature is often limited to either controlled clinical trial data with rigorous inclusion and exclusion criteria resulting in patient selection or to relatively short-term observational studies. In this study we have analyzed a real-life cohort of RVO patients with macular edema who received intravitreal corticosteroid injections with up to 3 years follow-up. Our results show that treatment with dexamethasone intravitreal implants (DII; Ozurdex, Allergan plc, Dublin, Ireland) can stabilize VA in the majority of eyes, despite the fact that most eyes had been pre-treated and were switched to DII treatment as a second-line therapy.

## Methods

All data in this retrospective study are from one center (University of Freiburg Medical Center). All eyes that fulfilled all of the following inclusion criteria were included: macular edema (ME) due to central retinal vein occlusion (CRVO) or branch retinal vein occlusion (BRVO), no contraindications for DII treatment, first DII injection between 2011 and 2016. Patients were enrolled consecutively if before mentioned criteria were applicable. In order to reflect the actual conditions in routine care, we did not apply specific exclusion criteria. The diagnosis of BRVO or CRVO with treatment-requiring macular edema was made based on fundoscopy, fluorescein angiography and optical coherence tomography (OCT). Decision for treatment with DII as well as the diagnosis BRVO or CRVO was made by the treating retinal specialist after informed consent of the patient. All patients were treated using a pro-re-nata regimen with a minimum interval of 3 months between DIIs. Patients with resolved ME were followed for at least 6 months with monthly OCT visits. In this retrospective observational study, we used aggregated patient data from routine examinations (real-life data). Data analysis was approved by the Ethics Board of the University of Freiburg Medical Centre (No. 26/15) and informed consent was obtained from all subjects included in this study. All experiments were performed in accordance with relevant guidelines and regulations. The methods of clinical data acquisition have been described previously^6,7^ and are briefly summarized here: all analyses reported in this study are per eye; VA was acquired on decimal charts and converted to ETDRS-equivalents for analysis. In contrast to controlled trials this is a real-life cohort, implying that a number of different operators collected VA as well as other measurements and that operators changed over the years. Autorefraction was performed before each VA exam and VA was recorded with autorefraction values as well as with the patient’s own glasses. If two VA measurements were available in the records, the better VA (autorefraction or own glasses) was used for analyses. Subjective correction of autorefraction measurements was not performed. For VA readings “hand motion” and “counting fingers”, we determined a numerical equivalent as described previously^8,9^. Central retinal thickness was determined by the built-in algorithm of the Heidelberg Spectralis OCT device (Heidelberg Engineering GmbH, Heidelberg Germany). Intraocular pressure was determined by non-contact tonometry and verified by Goldmann applanation tonometry if outside normal limits. Values in this manuscript represent either mean ± standard deviation or median ± IQR.

All eyes treated with DII were followed in this analysis until one of the following endpoints was reached: end of therapy (no further treatment required), loss to follow-up, switch to anti-VEGF treatment, switch to triamcinolone (TAC) treatment, termination of therapy (insufficient effect), termination of therapy (requested by patient) or vitrectomy. Data processing and descriptive statistics was done using GNU R and additional packages^10–12^.

## Results

**Figure 1:**
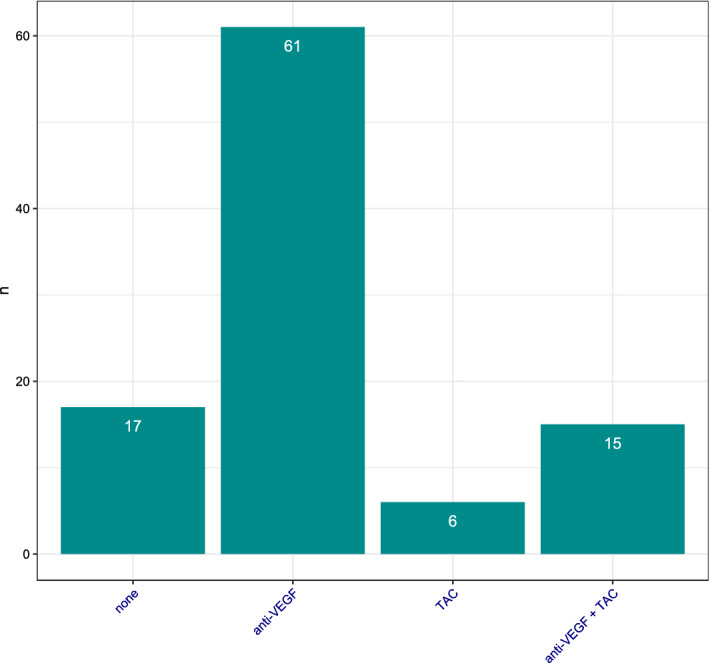
Pre-treatment status at baseline. The majority of eyes had pre-treatment with either anti-VEGF, triamcinolone (TAC) or both. Only 17 eyes (17%) received DII as first-line therapy.

In total, 99 eyes from 99 patients (48 females, 51 males) were included in our analysis. Patients’ median age at first DII treatment was 75.36 years (IQR 11.61). Most eyes were not treatment-naïve: 61 eyes (62%) had received anti-VEGF injections prior to DII, 6 eyes (6%) had received prior TAC and 15 eyes (15%) had received a pre-treatment with both anti-VEGF and TAC. Only 17 eyes (17%) received DII as first-line treatment (Fig. 1).

**Figure 2:**
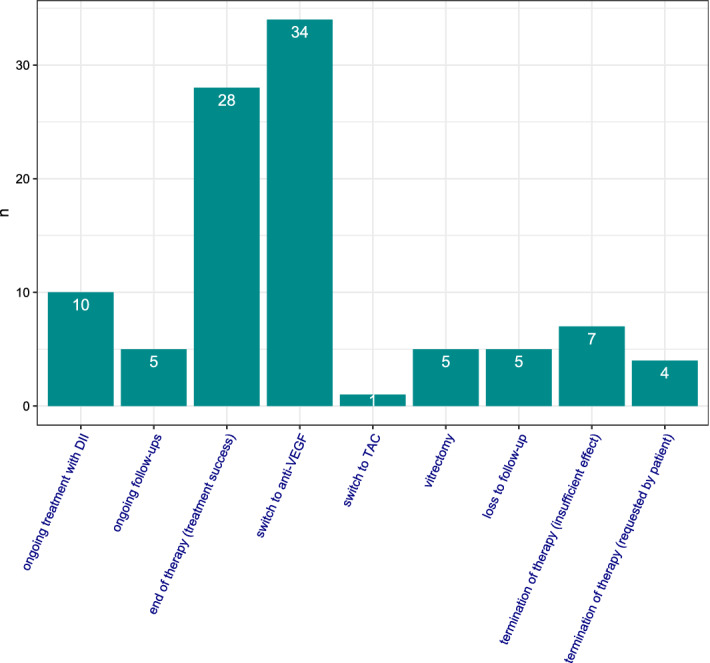
Outcome at end of observation. At the end of our observation window, ten eyes, (10%) were receiving ongoing DII treatment and five eyes (5%) had ongoing follow-up control visits. 28 eyes (28%) had been released from follow-up as treatment success. 34 eyes (34%) were switched to anti-VEGF therapy and five eyes (5%) required a vitrectomy. Five eyes (5%) were lost to follow-up and in 11 eyes (11%) further interventions were either considered not likely to yield benefit (seven eyes, 7%) or were stopped according to patient’s decision (four eyes, 4%).

Mean duration of follow-up in this study was 312 ± 310 days. The number of DII treatments ranged from 1 to 10 with a mean of 2.2 ± 2.1 injections. Follow-up ended for each individual eye when one of the defined endpoints was met (see “Methods”). Figure 2 shows that 28 eyes (28%) were released from follow-up as treatment success (i.e. resolved macular edema without rebound on follow-up OCTs for at least 3 months). An additional 15 eyes (15%) were still in follow-up (five eyes) or under continuous DII treatment (ten eyes). In a total of 47 eyes (47%) DII treatment did not yield the desired effect and patients were either stopped from further treatment (seven eyes), required vitrectomy (five eyes), or were switched to other intravitreal pharmacological treatments (35 eyes). A total of nine eyes were lost to follow-up either because the patient did not appear for further scheduled visits (five eyes), or the patient communicated his or her wish to end treatment (four eyes).

**Figure 3:**
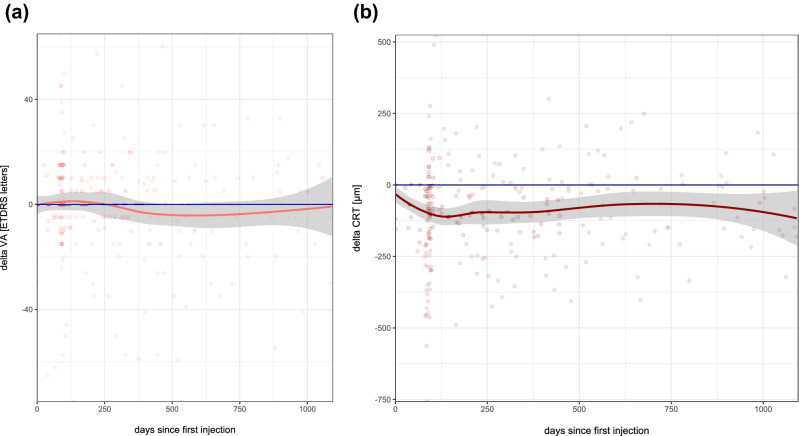
Visual acuity and central retinal thickness. Mean visual acuity stabilizes (**a**) and mean retinal thickness decreases (**b**) under DII treatment over three years. Note the high variability indicated by the individual dots in both plots. The gray area represents the 95% confidence interval.

The functional and morphologic results of DII treatment in our cohort are shown in Fig. 3. Mean visual acuity was maintained throughout the study phase with mean VA at baseline at 66.7 ± 23.5 letters and mean VA at last observation at 64.9 ± 28.3 which represents a marginal loss of 1.8 letters (Fig. 3a). Full OCT data was available for 95 eyes. Mean central retinal thickness decreased from 526 ± 179 µm at baseline to 431 ± 199 µm at the end of the observation period. Mean CRT reduction vs. baseline was 95 µm after 1 year, 66 µm after 2 years and 95 µm after 3 years (Fig. 3b).

**Figure 4:**
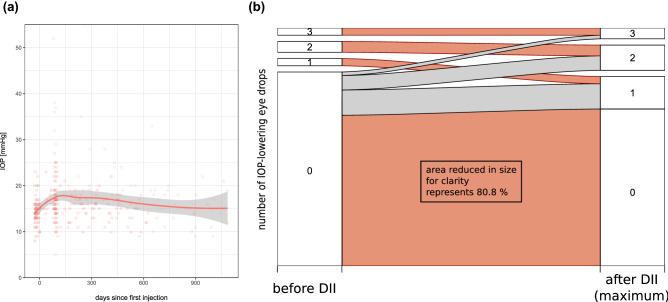
Intraocular pressure and IOP-lowering medication. Mean intraocular pressure (**a**) showed a slight increase around 4 weeks after initiation of DII treatment but remained between 15 and 20 mmHg at all time points. Individual eyes, however, showed significantly higher IOP levels. The use of IOP-lowering medication (**b**) remained relatively stable over time with more than 75% of patients not requiring IOP-lowering medication in all three years.

Two of the main concerns with DII treatment are elevation of intraocular pressure (IOP) and cataract formation. In our cohort, we observed a slight increase in mean IOP about 1 month after injection (Fig. 4a). For the whole observational period, mean IOP increased from 14.4 ± 3.1 mmHg at baseline to 17.1 ± 6.3 mmHg at last observation (complete IOP-data available for n = 67). The need for intraocular pressure-lowering eye drops shows a similar course over time: we observed a slight increase in the average consumption of IOP-lowering eye drops with a maximum at approximately 30 days after first DII injection. The majority of patients (well over 75% at all observation time points) did not require any IOP-lowering therapy during the entire follow-up period (Fig. 4b).

A total of three eyes had to undergo glaucoma surgery (two eyes: cyclophotocoagulation, one eye: minimally invasive glaucoma surgery). All three eyes had secondary glaucoma following retinal vein occlusion.

Regarding cataract formation, we counted 58 eyes in our cohort that were phakic at initiation of DII treatment and therefore at risk of cataract formation. Of these 58 phakic eyes, 13 eyes (22%) received cataract surgery during the follow-up period. With regard to the temporal distribution, cataract surgery was most frequently performed in the first and second year after DII injection. Only two eyes received cataract surgery in year 3 (Table 1).

**Table 1:** Cataract surgery.

	Cataract surgery (days after first DII treatment)	Age (years) at day of cataract surgery
1	56	72.4
2	99	74.4
3	149	72.4
4	220	89.3
5	227	84.6
6	490	59.2
7	528	67.0
8	575	85.6
9	580	78.3
10	581	79.2
11	598	82.9
12	609	71.5
13	615	66.1
14	907	63.6
15	945	80.0

In total, 15 of 58 eyes that were phakic at the time of first injection received cataract surgery within the first 3 years after their first DII injection. The table shows the temporal distribution of cataract surgery and age of the patient at surgery. Cataract surgery was most frequently performed in the first and second year after DII injection. Mean age of patients at cataract surgery was 75.1 (range 59–89). Mean BCVA before cataract surgery was 44.7 ± 21.1 letters. Mean IOP on the last visit before cataract surgery was 18.5 ± 5.6 mmHg.

**Figure 5:**
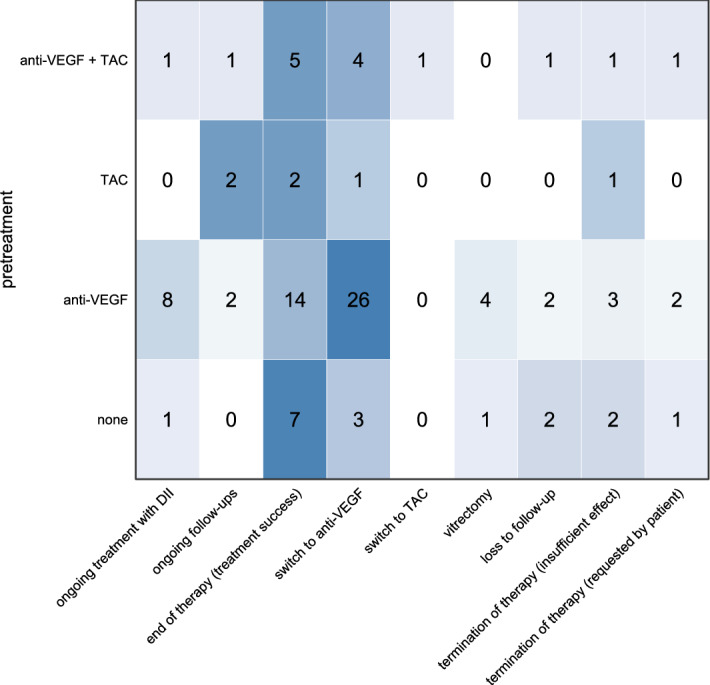
Correlation between pre-treatment and outcome. Most patients included in this study were pretreated with intravitreal injections, mostly anti-VEGF agents (see Table 2). No clear relationship was evident between the type and extent of pre-treatment and the outcome of DII treatment. Numbers in this matrix represent eyes while the color reflects the relative value per pre-treatment group.

Reflecting the real-world nature of our data, our patient group was heterogenous concerning previous treatment (Table 2). None of the pre-treatments was associated with an increased risk of treatment failure (Fig. 5).

**Table 2:** Demographic data and pre-treatments.

**Demographic data**	
Total number of eyes in study	n = 99
Male/female patients	Male n = 51 (52%) Female n = 48 (48%)
Median age [years (IQR)]	75.4 (11.6)
**Treatment before DII**	
Treatment-naïve	n = 17 (17%)
Anti-VEGF	n = 61 (62%)
Median number of anti-VEGF injections before DII per patient with VEGF pre-treatment (IQR)	6 (8)
TAC	n = 6 (6%)
Median number of TAC injections before DII per patient with TAC pre-treatment (IQR)	1 (0.75)
TAC+ anti-VEGF	n = 15 (15%)
Median number of TAC and anti-VEGF injecttions before DII per patient with VEGF+ TAC pre-treatment (IQR)	Anti-VEGF:11 (6.5) TAC: 1 (1)

## Discussion

In the present study, we investigated the effect of intravitreal therapy with a degradable dexamethasone intravitreal implant (DII) on medium-term visual acuity and retinal thickness outcomes in patients with macular edema after retinal vein occlusion. Data were collected from routine clinical records in a real-life setting and follow-up was up to 3 years.

Considering the fact that most patients (83%) in this study were not treatment-naïve at the time of first DII treatment, the overall VA results are not surprising. Mean VA could be stabilized but did not increase significantly. This may, to a large extent, be contributed to the fact that long-standing or frequently recurring edema has a lower potential for VA increase due to either macular ischemia or structural retinal damage or a combination of both. In this light, it is reassuring that almost one-third of the DII treated eyes were considered a treatment success (i.e. having fully resolved macular edemas). In a broader sense, if eyes that are still under follow-up examination or receiving continuous DII treatment are also considered a treatment success, then 43% of eyes can be considered as responding positively to DII treatment. At the same time, we observed 47% of eyes that did not respond sufficiently to DII treatment and were either switched (back) to other treatment modalities or not treated further. The fact that treatment naïve eyes may respond better to DII compared to switched eyes with potentially longer standing edema is supported by a study from Pielen et al.^13^.

In a real-life study similar to ours, Eter et al. found comparable VA results with BCVA gain ranging from 5.4 to 9.5 letters (depending on macular edema duration) 12 weeks after DII treatment^14^. Winterhalter et al. reported comparable VA results at month 3 in a real-life study of 48 eyes receiving DII for BRVO and CRVO and Chiquet et al. reported significant VA improvement when patients pre-treated with anti-VEGF were switched to DII treatment but not vice versa^15^. This group as well as Hanhart and Rozenman found a more pronounced reduction in CRT compared to our data^16,17^.

The observed data on IOP and cataract formation is overall reassuring. It must be noted, however, that similar to earlier studies^5,18–23^ we did observe increased IOP values around 4 weeks after initial treatment. Cataract surgery and elevated IOP occurred less frequently in our study compared to others^24^. The fact that the mean IOP curve returned back towards baseline values at later time points may be partially attributable to the fact that patients who showed a significant IOP increase following the first injection were unlikely to receive additional injections.

Limiting factors of our study are the retrospective and monocentric approach as well as the heterogeneity of both clinical presentations, pre-treatments and the confounding factor of multiple retina specialists being involved in clinical decision making. In addition, more than half of the eyes in our study were switched to other therapies, had termination of therapy due to insufficient effect or as requested by patient, or were lost to follow-up. This may have impacts on the results since these eyes were not available for further DII follow-ups. Strengths of this study include the fact that all available data were included and that the observed time span covers up to 3 years, thus providing representative medium term clinical data that has been underreported to date.

In summary, our study reports real-life effects of intravitreal dexamethasone treatment in patients with (predominantly pre-treated) macular edema following retinal vein occlusion. The reported data may help to give patients and health care providers guidelines on what can be expected with regard to VA and CRT development in this important indication.
